# *ZSWIM7* Is Associated With Human Female Meiosis and Familial Primary Ovarian Insufficiency

**DOI:** 10.1210/clinem/dgab597

**Published:** 2021-08-17

**Authors:** Sinéad M McGlacken-Byrne, Polona Le Quesne Stabej, Ignacio Del Valle, Louise Ocaka, Andrey Gagunashvili, Berta Crespo, Nadjeda Moreno, Chela James, Chiara Bacchelli, Mehul T Dattani, Hywel J Williams, Dan Kelberman, John C Achermann, Gerard S Conway

**Affiliations:** 1 Genetics and Genomic Medicine, UCL Great Ormond Street Institute of Child Health, University College London, London WC1N 1EH, UK; 2 Institute for Women’s Health, University College London, London WC1N 1EH, UK; 3 GOSgene, Genetics and Genomic Medicine, UCL Great Ormond Street Institute of Child Health, University College London, London WC1N 1EH, UK; 4 Department of Molecular Medicine and Pathology, University of Auckland, Auckland, New Zealand; 5 Developmental Biology and Cancer, UCL Great Ormond Street Institute of Child Health, University College London, London WC1N 1EH, UK; 6 Division of Cancer and Genetics, Genetic and Genomic Medicine, Cardiff University, Cardiff CF14 4AY, UK

**Keywords:** primary ovarian insufficiency, meiosis, ovary development, primary amenorrhea, delayed puberty, genetics, NGS

## Abstract

**Background:**

Primary ovarian insufficiency (POI) affects 1% of women and is associated with significant medical consequences. A genetic cause for POI can be found in up to 30% of women, elucidating key roles for these genes in human ovary development.

**Objective:**

We aimed to identify the genetic mechanism underlying early-onset POI in 2 sisters from a consanguineous pedigree.

**Methods:**

Genome sequencing and variant filtering using an autosomal recessive model was performed in the 2 affected sisters and their unaffected family members. Quantitative reverse transcriptase PCR (qRT-PCR) and RNA sequencing were used to study the expression of key genes at critical stages of human fetal gonad development (Carnegie Stage 22/23, 9 weeks post conception (wpc), 11 wpc, 15/16 wpc, 19/20 wpc) and in adult tissue.

**Results:**

Only 1 homozygous variant cosegregating with the POI phenotype was found: a single nucleotide substitution in zinc finger SWIM-type containing 7 (*ZSWIM7*), NM_001042697.2: c.173C > G; resulting in predicted loss-of-function p.(Ser58*). qRT-PCR demonstrated higher expression of *ZSWIM7* in the 15/16 wpc ovary compared with testis, corresponding to peak meiosis in the fetal ovary. RNA sequencing of fetal gonad samples showed that *ZSWIM7* has a similar temporal expression profile in the developing ovary to other homologous recombination genes.

**Main conclusions:**

Disruption of *ZSWIM7* is associated with POI in humans. *ZSWIM7* is likely to be important for human homologous recombination; these findings expand the range of genes associated with POI in women.

Primary ovarian insufficiency (POI) is an important and relatively common condition, affecting 1% of women and causing significant medical, psychosocial, and economic sequelae (1). It arises when a primary defect within the ovary results in ovary dysfunction and disruption of the resting follicle pool (2, 3). POI is diagnosed in women who present before the age of 40 years with amenorrhea of more than 4 months’ duration, estrogen deficiency, and raised FSH concentrations measured twice at least 1 month apart (1, 4). The majority of women with POI experience normal pubertal development and present in adulthood with secondary amenorrhea or oligomenorrhea (5-7). Adolescents presenting with primary amenorrhea and delayed puberty, accounting for 10% of presentations of this condition, represent the most severe end of the POI spectrum.

Establishing an underlying cause for a POI diagnosis can be difficult and in 50% to 80% of women a cause is not found (3, 8). Iatrogenic POI secondary to surgical oophorectomy, chemotherapy, or radiotherapy accounts for a substantial proportion (up to 30%) (8). Environmental toxins and an underlying autoimmune etiology have also been proposed (9, 10). So far, variants in more than 60 different genes have been associated with the pathogenesis of POI, with each gene responsible for only a small subset of cases (11). The advent of next-generation sequencing has proven to be a useful tool in expanding the number of known genetically mediated causes of POI. This has provided a genetic diagnosis for a proportion of affected women (~5%-30%) and allows targeted genetic counselling of patients and family members (5, 12). Notably, identified genes often relate to the known complex biological processes underpinning normal ovary development and function, including sex differentiation, oogenesis, folliculogenesis, and steroidogenesis.

Oogenesis is particularly critical for normal germ cell development and is dependent on meiosis, which encompasses a series of critically regulated processes that result in a diploid germ cell undergoing 1 round of DNA replication followed by 2 cell divisions to produce a haploid ovum capable of being fertilized. In females, meiosis commences in fetal life but is complete only after fertilization of a metaphase II oocyte during adult life. Meiosis begins with homologous chromosomes moving close to one another with the subsequent formation of the synaptonemal complex (SC). Cohesins form protein complexes to stabilize the SC. Homologous recombination follows, initiated by SPO11-mediated double-strand DNA breaks. Resected tails are bound by single-strand DNA-binding proteins to prevent reannealing and to recruit RAD51 and DMC1, which catalyze strand invasion and exchange (13, 14). DNA exchange intermediates are then joined via DNA repair mechanisms, which results in the formation of either crossover or noncrossover intermediates (15). Pathogenic variants in several meiosis genes have already been associated with POI. These include *SYCE1* and *SYCP3* (SC) (16, 17); *STAG3, REC8*, and *SMC1B* (cohesin complex) (18, 19); *MEIOB* and *BRCA2* (strand invasion) (20, 21); *MCM8, MCM9, MSH4, MSH5,* and *HFM1* (DNA repair; stabilization and intermediate processing) (22-26); *FANCM, BLM* (crossover regulation); and *MLH3* (crossover resolution) (27). Because meiosis is a complex process involving the coordinated interaction of many genes, it is likely that other factors involved in meiosis represent candidate genes for POI. Here, we associate an autosomal recessive pathogenic variant in the meiosis-associated gene zinc finger SWIM-type containing 7 (*ZSWIM7*), also known as *SWS1,* with primary ovarian insufficiency.

## Methods

### Participants and Genetic Analysis

A family with 2 sisters affected with POI was recruited as part of an ovarian dysgenesis research project at University College London Hospitals (08/H071/69). In an attempt to find a genetic cause of POI, 2 affected and 2 unaffected members (mother and sibling) of the family underwent whole-genome sequencing.

After obtaining informed consent, genomic DNA was isolated from frozen whole blood using a QIAamp DNA Blood Mini kit (Qiagen). Library preparation and whole-genome sequencing were performed at BGI Genomics (Shenzhen, China) using the BGISEQ-500 platform at ~30× coverage. Sequencing reads were aligned with Burrows-Wheeler Aligner v0.7.17 to human genome build 38 (GRCh38.p1), not including alternate assemblies (GCA_000001405.15_GRCh38_no_alt_analysis_set.fna), and read duplicates were marked with Sambamba (28, 29). Variant calling across the exonic regions with 100-bp padding was performed using Genome Analysis Toolkit v4.0.3.0 according to the best practices workflow for joint (multisample) calling (30-32). Variant filtering was performed through the use of Ingenuity Variant Analysis software (Qiagen). We focused our analysis on coding and splice region (7 bases into an intron) variants with read depth ≥ 5 that are not present or rare (allele frequency ≤ 0.01%) in the Genome Aggregation Database (gnomAD v2.1.1, Cambridge, MA; https://gnomad.broadinstitute.org, accessed March 2021 (33)) using an autosomal recessive mode of inheritance because of consanguinity in the family. Synonymous changes were excluded, unless they were predicted to affect splicing using MaxEntScan. Sanger sequencing was used to verify the presence of the variant and to perform segregation analysis. The target region was amplified using primers (forward 5′-CAAGTTGGGGCAAAAGCCTT-3′ and reverse 5′-CCTTTGGGCAAGTTACTGAGG- 3′). PCR products were purified with ExoSap and sequenced using Big Dye Terminator Cycle Sequencing Kit v3.1 (Life Technologies, Foster City, CA). Result electropherograms were analyzed with Geneious software (Biomatters Ltd., Auckland, New Zealand).

The American College of Medical Genetics variant classification guidelines and in silico software prediction tools Decipher, PROVEAN, MutationTaster, and CADD scoring were used to assess variant pathogenicity (34-38).

### Quantitative Reverse Transcriptase PCR Analysis of *ZSWIM7*

Quantitative reverse transcriptase PCR (qRT-PCR) was used to study the expression of *ZSWIM7* during fetal gonadal development. Human fetal tissue samples were obtained with ethical approval (REC references 18/LO/0822; 18/NE/0290) and informed consent from the Human Developmental Biology Resource (http://www.hdbr.org). Four ovary and testis samples were included at each of 5 developmental stages: Carnegie Stage (CS) 22/23, 7.5 to 8 weeks postconception (wpc), 9 wpc, 11 wpc, 15 to 16 wpc, and 19 to 20 wpc. Four adult ovary (catalog number CS500008, Origene) and 4 adult testis samples (catalog number CS502309, Origene) were also included. RNA was quantified using a NanoDrop 1000 spectrophotometer (Thermo Fisher Scientific) and reverse transcribed using the SuperScript III Reverse Transcriptase kit (Thermo Fisher Scientific). qRT-PCR was performed using Taqman Fast Advanced MasterMix (Applied Biosystems) and TaqMan assays (*ZSWIM7:* Hs04984973_m1) on the ABI StepOne Plus System (Applied Biosystems). The relative expression of *ZSWIM7* was calculated using the comparative Ct method and *GAPDH* (Hs02786624_g1) was used as a housekeeping gene control. Experiments were conducted in triplicate on 3 occasions. Data are expressed as mean ± SEM. Two-way ANOVA and independent samples *t* tests were used for statistical analysis (GraphPad Prism v9.0.0). Further analysis of *ZSWIM7* expression in adult tissues was performed using GTEx data (v.8; accessed March 2021), the Human Protein Atlas (v20.1; accessed March 2021), and FANTOM5 (accessed March 2021).

### RNA Expression in Human Fetal Gonad Development

The expression of *ZSWIM7* and associated DNA repair genes in fetal development was studied using bulk RNA sequencing. Five ovary (4 at 15-16 wpc), 5 testes, and 2 46,XX control tissues were included at each of 4 developmental stages: CS22, 9 wpc, 11 wpc, and 15 to 16 wpc. RNA was extracted using the AllPrep DNA/RNA Mini Kit (Qiagen) using the kit manufacturer’s instructions. Libraries were prepared using the KAPA RNA HyperPrep Kit with RiboErase (Illumina) and sequenced with the Illumina HiSeq 4000 platform using 2 × 75-bp paired-end sequencing kit. Quality control of sequencing reads was performed using FastQC (Babraham Bioinformatics) and the reads were aligned to human genome build 38 (GRCh38.p1) using STAR aligner (v2.5.2a) (39, 40). featureCounts (Subread package) and DESeq2 (Bioconductor) were used for gene expression quantification and differential gene expression analysis, respectively (41, 42). The Benjamini-Hochberg approach was used to adjust for multiple testing with cutoff adjusted *P* values of 0.05 (43).

## Results

### POI Pedigree

**Figure 1.**
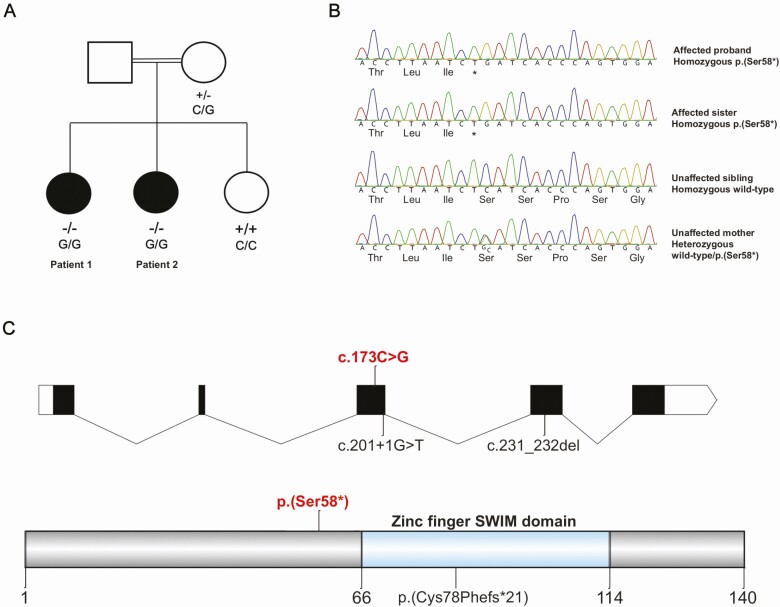
(A) Affected kindred with the *ZSWIM7* variant (NM_001042697.2:c.173C > G, p.(Ser58*)). Solid symbols indicate affected family members. Genotype is indicated underneath tested family members. (B) Sanger sequencing of the affected proband, an affected sister, an unaffected sister, and unaffected mother. NC_000017.11g:g.15987294G > C; NM_001042697.2:c.173C > G; NP_001036162.1:p.(Ser58*). (C) Domains of the human ZSWIM7 protein. The p.(Ser58*) stop-gain variant reported in this study is proximal (N-terminal) to the zinc finger SWIM domain. Previously reported *ZSWIM7* variants (associated with male infertility) are also indicated (homozygous splice variant c.201 + 1G > T; homozygous frameshift variant c.231_232del; p.(Cys78Phefs*21)).

Two sisters from a consanguineous pedigree presented in adolescence with absent puberty and primary amenorrhea (Fig. 1A). Their parents were first cousins originating from Turkey. Both had raised gonadotropin and low estradiol concentrations consistent with a diagnosis of POI (Table 1). Extended characterization for the etiology of ovarian insufficiency in both girls demonstrated 46,XX karyotypes and negative Fragile X screening (*FRAXA* premutation analysis). Both were normotensive at presentation. Pelvic ultrasound did not identify ovaries. There was no history of previous ovarian surgery nor chemo-/radiotherapy. Both patients were commenced on estrogen replacement and subsequently progressed through puberty as expected, with normal breast development, development of secondary sexual characteristics, and menarche. Their parents had normal fertility and there was no family history of delayed puberty nor infertility in the extended family. A sister was unaffected and developed in puberty normally.

**Table 1.** Clinical characteristics of 2 women with primary ovarian insufficiency

	Patient 1	Patient 2
Menarche	Primary amenorrhea	Primary amenorrhea
Age at presentation, y	15	12
Pubertal induction required	Yes	Yes
FSH at diagnosis, IU/L (follicular range: 3.5-12.5)	94.8	77.8
LH at diagnosis, IU/L (follicular range: 2.4-12.6)	17.2	14.3
E2 at diagnosis (follicular range: 98-571 pmol/L; undetectable < 20 pmol/L)	Undetectable	Undetectable
Pubertal stage at diagnosis	B2 P1 A1	B1 P1 A1
Karyotype	46,XX	46,XX
Ovaries on imaging at diagnosis (normal range 5-7 cm^3^)	Small prepubertal uterus; streak ovaries	Small prepubertal uterus; no ovaries seen
Fragile X genetic testing (FRAXA analysis)	Negative	Negative
Weight, kg	65.9	Not available
Height, cm	1.65	Not available
Body mass index, centile	92nd	-

### Homozygous Stop Gain Variant Identified in *ZSWIM7*

Analysis of coding and splice region variants using a model consistent with autosomal recessive inheritance revealed only 1 biallelic variant that cosegregated with the primary ovarian insufficiency phenotype.

This single nucleotide substitution occurred in *ZSWIM7* (NC_000017.11: g.15987294G > C, NM_001042697.2: c.173C > G) and is predicted to result in a stop-gain change at codon 58, p.(Ser58*). The 2 affected sisters were homozygous for this variant (allele fraction 1.0; read depths 65 and 54), whereas the mother was heterozygous (read depth 43, allele fraction 0.53). DNA from the father of the proband was not available (Fig. 1A). The presence of a larger structural variant, such as a deletion, at the locus was not evident from the mapped sequencing reads. The *ZSWIM7* variant was confirmed with Sanger sequencing (Fig. 1B). The variant is novel and has not been previously documented or reported in public databases (gnomAD and dbSNP). There is no *ZSWIM7-*associated phenotype in OMIM (MIM 614535). *ZSWIM7* encodes for a protein known to be important for meiotic homologous recombination during prophase I (Fig. 1C) (44). No homozygous loss-of-function *ZSWIM7* variants are reported in the gnomAD database. According to Decipher, the *ZSWIM7* transcript carrying a premature termination codon at position 58 is predicted to undergo nonsense mediated decay (36). Using the American College of Medical Genetics classification system, this variant is classed as pathogenic (1a) because: (1) it is a stop-gain mutation subject to nonsense-mediated decay (very strong evidence: PVS1); (2) there are existing animal models supportive of a damaging effect of disruption in this gene (strong evidence: PS3); (3) it cosegregates with disease in family members (supporting evidence: PP1); and (4) the variant is predicted pathogenic using in silico tools (supporting evidence: PP3; Table 2).

**Table 2.** Analysis of the c.173C > G variant

	Variant
Gene symbol	*ZSWIM7*
Full gene name	Zinc finger swim domain-containing protein 7
Position	Exonic
Cytoband	17p12
Region	Chr17:15987294
Transcript identification	NM_001042697.2
Exon involved	Exon 3
cDNA variant	c.173C > G
Protein variant	p.S58*
Translation impact	Stop gain
Genotype	Homozygous
CADD score	39 (pathogenic)
PROVEAN score	-7.28 (deleterious)
MutationTaster	Disease causing
DECIPHER	Predicted nonsense-mediated decay
Allele frequency in gnomAD	0
American College of Medical Genetics classification	Predicted pathogenic (1a)

### Expression of *ZSWIM7* in Human Ovary and Testis

**Figure 2.**
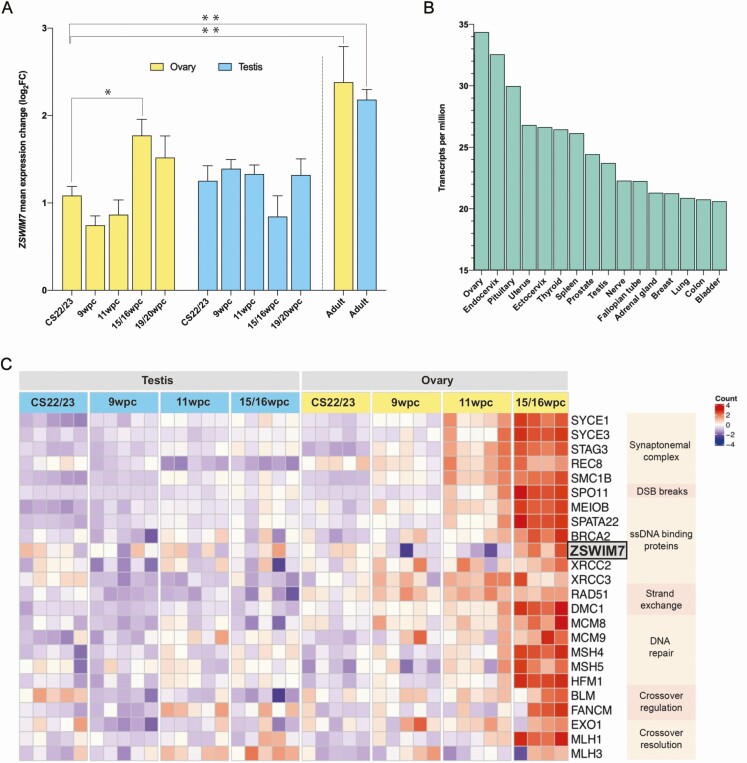
(A) qRT-PCR mean expression (log_2_) of *ZSWIM7* in various tissues compared with reference (GAPDH) and relative to the expression of *ZSWIM7* in a Carnegie Stage (CS) 22-ovary sample. Four fetal ovary and fetal testis tissue samples were included at each of the following stages: CS22/CS23, 9 wpc, 11wpc, 15 to 16 wpc, and 19 to 20 wpc. Bar heights indicate mean expression. Error bars indicate mean ± SEM. Independent samples *t* tests and 1-way ANOVA testing were used to assess differences in *ZSWIM7* mean expression change between tissue stages (*P < 0.05; **P < 0.01). There was significantly higher *ZSWIM7* expression in the 15 to 16 wpc ovary compared with the 15 to 16 wpc testis (*P* = 0.02). There was significantly higher *ZSWIM7* expression in the adult ovary compared with the CS22/23 ovary (*P* = 0.004) and in the adult testes compared with the CS22/23 testes (*P* = 0.003) compared with the CS22/23 testes. (B) *ZSWIM7* expression across adult tissues from the GTEx database (v8). Data are expressed in transcripts per million. (C) Heatmap representing differential gene expression of key meiotic genes during prophase I across 4 developmental timepoints (CS22/23, 9 wpc, 11 wpc, 15-16 wpc). The intensity of gene expression is indicated by a color scale: violet for lowest expression and red for highest expression. *ZSWIM7* is highlighted in gray. GAPDH, glyceraldehyde 3-phophate dehydrogenase; qRT-PCR, quantitative reverse transcriptase PCR; wpc, weeks postconception.

To investigate a role for *ZSWIM7* in female meiosis, the expression of this gene was quantified by qRT-PCR of human gonadal tissue at critical stages of fetal gonad development, including sex differentiation, germ cell expansion, and meiotic entry (CS22/23 [7.5-8 wpc], 9 wpc, 11 wpc, 15-16 wpc, and 19-20 wpc) (Fig. 2A; left panel). There was higher expression of *ZSWIM7* in the 15 to 16 wpc ovary compared with testis, corresponding with the peak onset of meiosis in the fetal ovary.

qRT-PCR analysis of adult ovary and testis showed relatively strong expression in the adult testis, where meiosis is actively occurring, but also in the adult ovary (Fig. 2A; right panel). This observation was supported following analysis of publicly available RNA expression datasets (GTEx v.8; Human Protein Atlas v20.1; FANTOM5), where *ZSWIM7* was found to be expressed in adult ovarian tissue (45-47). *ZSWIM7* expression across several tissues is displayed in Fig. 2B. These data suggest a potential long-term role for *ZSWIM7* beyond the developmental period.

### *ZSWIM7* Shows Similar Temporal Expression to Other Homologous Recombination Genes in the Human Fetal Ovary

To investigate the temporal expression of *ZSWIM7* during human fetal gonad development further, a time-series analysis using RNA sequencing of groups of ovary and testis samples between CS22/23 and 15 to 16 wpc was undertaken (Fig. 2C). *ZSWIM7* showed a peak of increased expression in the ovary at 15 to 16 wpc compared with CS22 (FC 1.25, *P*adj < 0.05), 9 wpc (FC 1.46, *P*adj < 0.05), and 11 wpc (FC 1.48, *P*adj < 0.05), consistent with an increase in meiosis across this timeframe. Higher expression of *ZSWIM7* was observed in the 15 to 16 wpc ovary compared with the testis at the same stage (FC 1.27, *P*adj = 0.05).

Finally, the developmental time-series RNAseq dataset was used to study human fetal gonadal expression of genes postulated to interact with *ZSWIM7*, as well as genes known to be important for prophase I of meiosis (Fig. 2C). Consistent upregulation of these genes was seen coinciding with peak meiosis in the 15 to 16 wpc ovary, when compared with testis and earlier premeiotic ovarian tissue (Fig. 2C).

## Discussion

Here, we report a homozygous, stop-gain mutation in *ZSWIM7,* an emerging key factor in the meiotic pathway, in 2 sisters with early-onset POI from a consanguineous family. The predicted pathogenic variant is proximal to the zinc finger domain of ZSWIM7 and likely results in nonsense-mediated decay or protein truncation with loss of this critical domain.

**Figure 3.**
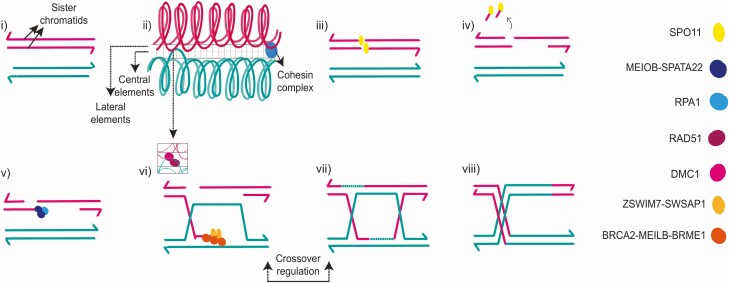
Genes associated with primary ovarian insufficiency (POI) and their relationship with the stages of meiosis I prophase. Genes associated with POI are italicized and in bold in this legend text. (i) Meiosis I begins with homologous chromosomes moving physically close to one another. (ii) The synaptonemal complex (SC) forms between closely apposed homologous chromosomes, composed of central ***(SYCE1)*** and lateral ***(SYCE3)*** elements and stabilized by cohesin complexes ***(STAG3, REC8, SMC1B)*** surrounding the chromatid. (iii) Homologous recombination is initiated by double strand DNA breaks (DSBs) ***(SPO11).*** (iv) DSBs are recognized, partially degraded, and act as substrate for homology searching ***(EXO1).*** (v) Resected DNA ends are recognized and bound by single-strand DNA (ssDNA) binding proteins ***(MEIOB)*** which prevent reannealing. (vi) Downstream binding proteins are then localized to the single-strand tails ***(BRCA2)***, which recruit strand-exchange nucleoprotein filaments ***(RAD51),*** stabilize and remodel the growing DNA filament ***(ZSWIM7),*** and catalyze strand invasion, exchange, and homologous pairing. (vii) DNA exchange intermediates are further processed and joined via DNA repair ***(MCM8, MCM9, MSH4, MSH5, HFM1).*** Crossover (pictured) or noncrossover (not pictured) intermediates result. Crossover formation is tightly regulated ***(BLM, FANCM).*** (viii) Resolution of crossover and noncrossover intermediates ***(MLH1, MLH3, EXO1)*** complete the homologous recombination process. Prophase I concludes with diplotene and diakinesis.

Functional studies have demonstrated ZSWIM7, also known as SWS1, to be a key regulator of meiotic homologous recombination. ZSWIM7/SWS1 interacts with SWSAP1 to form the highly conserved human Shu complex (48-50). This complex is required to regulate the recruitment of the strand-exchange protein RAD51 and its homolog DMC1 to meiotic intermediates during homologous recombination (Fig. 3). Loss of this complex in yeast, *Caenorhabditis elegans*, and in mice results in preserved viability but impaired fertility in males and females (44, 51-56). For example, *Sws1/Swsap1* knock-out mice are grossly morphologically normal but show severe defects in meiotic progression in both sexes with reduced Rad51 and Dmc1 foci formation (44). The weights of both ovary and testis are reduced and histologically show fewer postmeiotic germ cells, evidence of germ cell apoptosis, and higher numbers of early prophase meiocytes (44). Germ cells from *Zswim7*-depleted mice can form double-stranded DNA breaks normally, but demonstrate synapsis defects and an increase in MEIOB foci (44, 57). Loss of the BRCA2 C-terminus aggravates the phenotype of Sws1/Swsap1-deficient mice, with hybrid knockout models displaying increased synapsis defects. Furthermore, disruption of DMC1 in mice results in synaptic defects similar to those seen in *Zswim7* knockout animals (58, 59). These findings suggest that the Sws1/Swsap1 complex is essential for homologous recombination and that abnormal ZSWIM7 results in aberrant progression through prophase I (Fig. 3) (44). They also suggest that *ZSWIM7* may have overlapping biological roles with other homologous recombination genes.

Several homologous recombination genes have been implicated in the pathogenesis of POI and azoospermia, including some which are postulated to interact with *ZSWIM7* as described previously. Missense variants and single-nucleotide polymorphisms in *RAD51* have been associated with human ovarian dysgenesis and with lower age of menopause, respectively (60, 61). A homozygote mutation in *XRCC2,* a paralog of *RAD51,* was identified within a consanguineous pedigree in which males presented with azoospermia and infertility (62). Biallelic *BRCA2* variants, within the C-terminus domain, have been reported in both familial and sporadic POI (21, 63). Reduced foci of DMC1 and RAD51 have been noted in fibroblasts of patients with POI and homozygote *BRCA2* variants (21, 44). Recently, a homozygous splice variant (c.201 + 1G > T) (64) and a homozygous frameshift variant (c.231_232del; p.(Cys78Phefs*21)) (57, 64) in *ZSWIM7* itself have been shown to be associated with human male infertility in 4 unrelated patients (Fig. 1C). Both variants likely result in either nonsense mediated decay or a truncated protein missing the zinc-finger binding domain of ZSWIM7.

Despite a clear emerging role for ZSWIM7 in mammalian meiosis, the expression of *ZSWIM7* during human gonadal development has not been explored. Here, we demonstrate *ZSWIM7* expression in the developing human ovary with highest expression at 15 to 16 wpc that corresponds with the known peak of meiotic activity (65). We also show increased *ZSWIM7* expression in the adult testis and ovary. Significant *ZSWIM7* expression in the adult testis is expected given the known postpubertal timing of male meiosis, but the relatively high expression of *ZSWIM7* in the adult ovary, and well as degree of ubiquitous expression elsewhere, suggests that *ZSWIM7* is involved in recombinational DNA repair pathways outside of meiosis (66). We also demonstrate that *ZSWIM7* is expressed during the onset of human meiosis in fetal life and that this expression coincides with the expression of other known meiotic genes within the prophase I pathway.

To our knowledge, the stop-gain variant identified here in *ZSWIM7* is the first variant within this gene to be associated with human POI. Our association of *ZSWIM7* with POI suggests that disruption of this gene can result in both POI and male factor infertility, which has important clinical implications when counseling patients and their families. *Zswim7 (Sws1)/Swsap1* mutant mice reproduce the infertility phenotype, demonstrating marked meiotic abnormalities. This emphasizes the value of drawing on knowledge from existing model systems when clarifying human biology. In the future, knowing the specific genetic cause of a POI diagnosis may help in the development of personalized targeted treatments, especially in males in whom the onset of meiosis occurs only after puberty and in adulthood.

Taken together, these data provide evidence for a role for *ZSWIM7* in human female meiosis, implicate it in the pathogenesis of POI, and emphasize the importance of genes associated with homologous recombination and specifically meiosis prophase I in this condition. A broader mechanistic understanding of POI can be gained from considering meiotic genes as functional partners and this approach could extend the list of potential candidate genes for POI.

## Data Availability

Restrictions apply to the availability of genome sequencing data generated and analyzed during this study to preserve patient confidentiality. The corresponding author will on request detail the restrictions and any conditions under which access to some data may be provided. RNA sequencing data generated and analyzed during this study are included in the data repository BioStudies under accession number S-BSST693 at https://www.ebi.ac.uk/biostudies/.

## Abbreviations

CS: Carnegie Stage
gnomAD: Genome Aggregation Database
POI: primary ovarian insufficiency
qRT-PCR: quantitative reverse transcriptase PCR
SC: synaptonemal complex
wpc: weeks post conception
ZSWIM7: zinc finger SWIM-type containing 7

## Additional Information

**Disclosures:** The authors have nothing to disclose.
